# *Dayao* gen. n. of the subtribe Tyrina (Coleoptera, Staphylinidae, Pselaphinae) from South China

**DOI:** 10.3897/zookeys.141.1948

**Published:** 2011-10-28

**Authors:** Zi-Wei Yin, Li-Zhen Li, Mei-Jun Zhao

**Affiliations:** Department of Biology, College of Life and Environmental Sciences, Shanghai Normal University, Shanghai, 200234, P. R. China

**Keywords:** Staphylinidae, Pselaphinae, Tyrina, new genus, new species, taxonomy, Dayao Mountain, Guangxi, South China

## Abstract

*Dayao pengzhongi* **gen. et sp. n.** is described and illustrated based on the material collected in Guangxi Province, South China. The genus is placed in the oriental ‘*Pselaphodes* *complex*’ of genera of the subtribe Tyrina and its taxonomic placement is discussed.

## Introduction

According to the most recent catalog of the tribe Tyrini (Hlaváč and Chandler 2005), 13 genera of the subtribe Tyrina have been known from the Oriental region. Eight of 13 genera centered on the genus *Pselaphodes* Westwood grouped as the ‘*Pselaphodes* complex’ of genera (Hlaváč 2002: 283): *Indophodes* Hlaváč, *Labomimus* Sharp, *Lasinus* Sharp, *Linan* Hlaváč, *Nomuraius* Hlaváč, *Paralasinus* Hlaváč & Nomura, *Pselaphodes* Westwood and *Taiwanophodes* Hlaváč. Determination of the generic placement within the group is usually based on the form of maxillary palpi, in the combination with the foveal pattern on the head, pronotum and thorax and the relative length of the scape.

Recently a small series of pselaphine was collected at Dayao Mountain in the Guangxi Province, South China. The pselaphines were identified as a member of the ‘*Pselaphodes* complex’ upon an examination of morphological characters, but eventually found not matching any current generic conception of a known genus. The aim of this paper is to describe the new genus and species in detail, provide illustrations of the diagnostic characters, and discuss its taxonomic placement.

## Material and methods

All specimens were collected from the leaf litter of the forest floor by sifting. They were killed with ethyl acetate and then dried. Dissections were done in 75% ethanol. The genital organs and other dissected parts were mounted in Euparal (Chroma Gesellschaft Schmidt, Koengen, Germany) on plastic slides that were placed on the same pin as the specimen. Photos were taken by a Canon EOS 40D Camera mounted with an MP-E 65 mm Macro Photo Lens; line drawings were made using Adobe Illustrator CS2 based on the photos taken by a Canon G9 Camera mounted on an Olympus CX31 microscope.

A slash (/) is used to separate lines on the same label, and a double slash (//) is used to separate different labels on the same pin.

The following acronyms are used in the text:

ALlength of the abdomen;
            

AWmaximum width of the abdomen;
            

BLlength of the body (= HL + PL + EL + AL);
            

ELlength of the elytra, measured along sutural line;
            

EWmaximum width of the elytra;
            

HLlength of the head, measured from the anterior clypeal margin to the occipital constriction;
            

HWwidth of the head across eyes;
            

PLlength of the pronotum along midline;
            

PWmaximum width of the pronotum;
            

SHNUShanghai Normal University, Shanghai, P. R. China.
            

The type series are deposited in the Insect Collections of Shanghai Normal University, Shanghai, China (**SNUC**).

The terminology of foveal system follows Chandler (2001), except for using ‘ventrite’ instead of ‘sternite’.

## Taxonomy

### Dayao

Yin, Li & Zhao gen. n.

urn:lsid:zoobank.org:act:12861B33-83EB-466C-BF28-FDE4F6F6C17B

http://species-id.net/wiki/Dayao

Figs 1 [Fig F2] 13 

#### Type species.

 *Dayao pengzhongi* Yin, Li and Zhao, here designated. Gender masculine.

#### Diagnosis.

 Head and pronotum finely punctate. Head with vertexal foveae small, lacking frontal foveae; maxillary palpi with apical three segments elongate, each basally pedunculate and strongly protrude laterally. Pronotum with median and lateral antebasal foveae, lacking antebasal sulcus. Median metaventral fovea absent. Abdomen with tergite IV longest.

#### Description.

Length 2.95–2.99 mm. Head with narrow, long and prominent frontal rostrum, antennal tubercles faint; small vertexal foveae nude, with median carina between foveae; lacking postantennal notches and lateral postantennal pits; antennae with 11 antennomeres, antennal club formed by three enlarged apical antennomeres; maxillary palpi (Fig. 9) elongate, lateral projections of antennomeres II–IV each setose at their apices, fourth palpomeres with apical palpal cones; with lateral genal spines; gula flat, gular foveae close in median impression.

Pronotum with nude median and setose lateral antebasal foveae; lateral procoxal foveae present.

Each elytron with two basal foveae; discal stria extending from second basal fovea and exceeding elytral midpoint.

Thorax with median and lateral mesoventral foveae; lateral mesocoxal foveae present; lacking median metaventral fovea, metaventral apex broad and shallowly notched medially.

Legs with tarsomeres simple, third tasomeres about 0.75 times as long as second tarsomeres.

Abdomen with tergite IV (visible tergite I) longer than V–VII combined; tergite IV with deep basal sulcus connecting basolateral foveae, lacking mediobasal fovea in sulcus; with long discal carinae; tergites V–VII each with basolateral foveae. Sternite IV largest, longer than V–VII combined, with deep basal sulcus densely covered by short setae.

Males with antennomeres IX, pronotum and protibiae modified. Aedeagus with median lobe asymmetric; parameres long and symmetric; dorsal diaphragm oval.

#### Distribution.

 A single species in known from Dayao Mountain, Guangxi Province, South China.

#### Comparative notes.

*Dayao* is placed near the genera of *Pselaphodex* complex with simple, linear tarsomere II not strongly bilobed, only slightly extending beneath tarsomere III: *Paralasinus* Hlaváč & Nomura, *Lasinus* Sharp, *Linan* Hlaváč, *Indophodes* Hlaváč, *Labomimus* Sharp and *Pselaphodes* Westwood*.* Among these genera, *Dayao* can be readily separated from *Paralasinus* and *Lasinus* by the clearly asymmetrical palpomeres II–IV, which are simple in both genera. *Dayao* is separated from *Indophodes* and *Labomimus* by the lack of a frontal and a median metaventral fovea; from *Pselaphodes* by the lack of a frontal fovea, the indistinct pronotal lateral antebasal foveae and the longer scape. The lack of a median metaventral fovea, the pronotum being finely punctate and the basal carinae of the tergite IV being much longer separate *Dayao* from *Linan* in which the median metaventral fovea is present, the pronotum is roughly and densely punctate and the basal carinae are much shorter.

#### Remarks.

The published key to the world genera of Tyrini (Hlaváč and Chandler 2005) may be modified as the following to accommodate the new genus:

**Table:** 

18(16)	Head with vertexal foveae indistinct, lacking frontal fovea; lacking median longitudinal sulcus on the pronotal disc	18a
–	Head with distinct setose vertexal and frontal foveae; pronotum with median longitudinal sulcus on the disc variably present	19
18a	Head and pronotum roughly punctate; median metaventral fovea present; discal carinae of tergite IV short or indistinct. (Northwestern Thailand; China: Yunnan, Zhejiang, Hainan, Anhui, Guizhou and Jiangxi Provinces)	*Linan* Hlaváč
–	Head and pronotum finely punctate (Figs 4–5); median metaventral fovea absent (Fig. 6); long discal carinae of tergite IV distinct (Fig. 1). (China: Guangxi Province)	*Dayao* Yin, Li & Zhao, gen. n.

#### Etymology.

The generic name is taken from the collection site of the type series, Dayao Mountain.

### Dayao pengzhongi

Yin, Li & Zhao sp. n.

urn:lsid:zoobank.org:act:CA2D75C0-637D-439A-96A3-B0A9585E3601

http://species-id.net/wiki/Dayao_pengzhongi

Figs 1 [Fig F2] 13 

#### Type material.

 (1 ♂, 3 ♀♀)**.** Holotype: ♂, labelled ‘**CHINA:** Guangxi Prov. / Laibin City, Jinxiu County / Dayao Mt., 7 km / 1,200–1,400 m, 22.vii.2011 / Z. Peng leg. // [red label] HOLOTYPE / *Dayao pengzhongi* Yin et al. / SHNU Collections’. Paratypes: 3 ♀♀, same label data as holotype, except ‘23.vii.2011 / Z.W. Yin & J.Y. Hu leg.’, all bear the following label: ‘[yellow label] PARATYPE / *Dayao pengzhongi* Yin et al. / SHNU Collections’.

#### Description.

**Figures 1.**
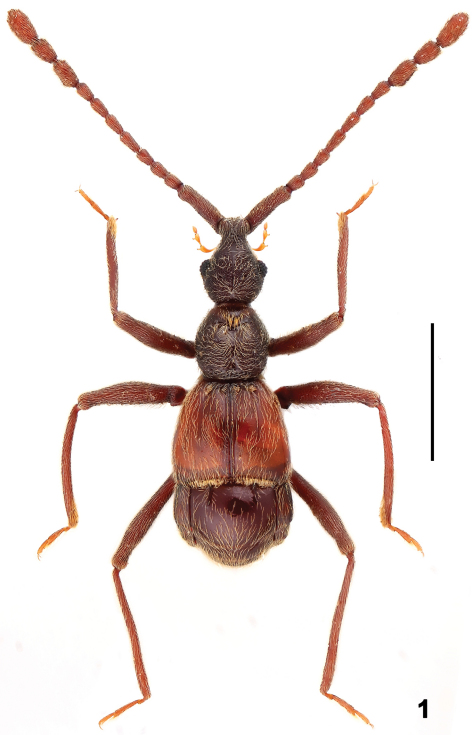
Male habitus of *Dayao pengzhongi*, holotype. Scale: 1.0 mm.

**Figures 2–7. F2:**
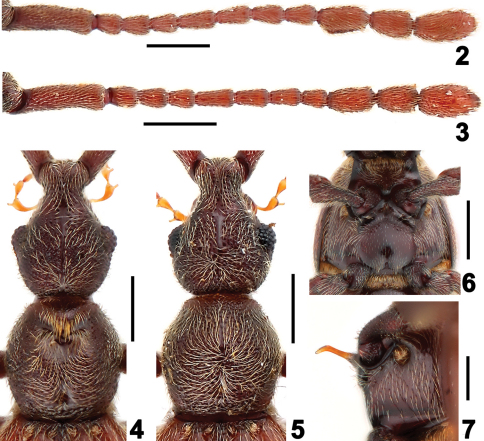
Details of *Dayao pengzhongi*. **2** male right antenna **3** same, female **4** male head and pronotum **5** same, female **6** male meso- and metathorax **7** male metaventral process, in lateral view. Scales: 0.3 mm.

**Figures 8–13.**
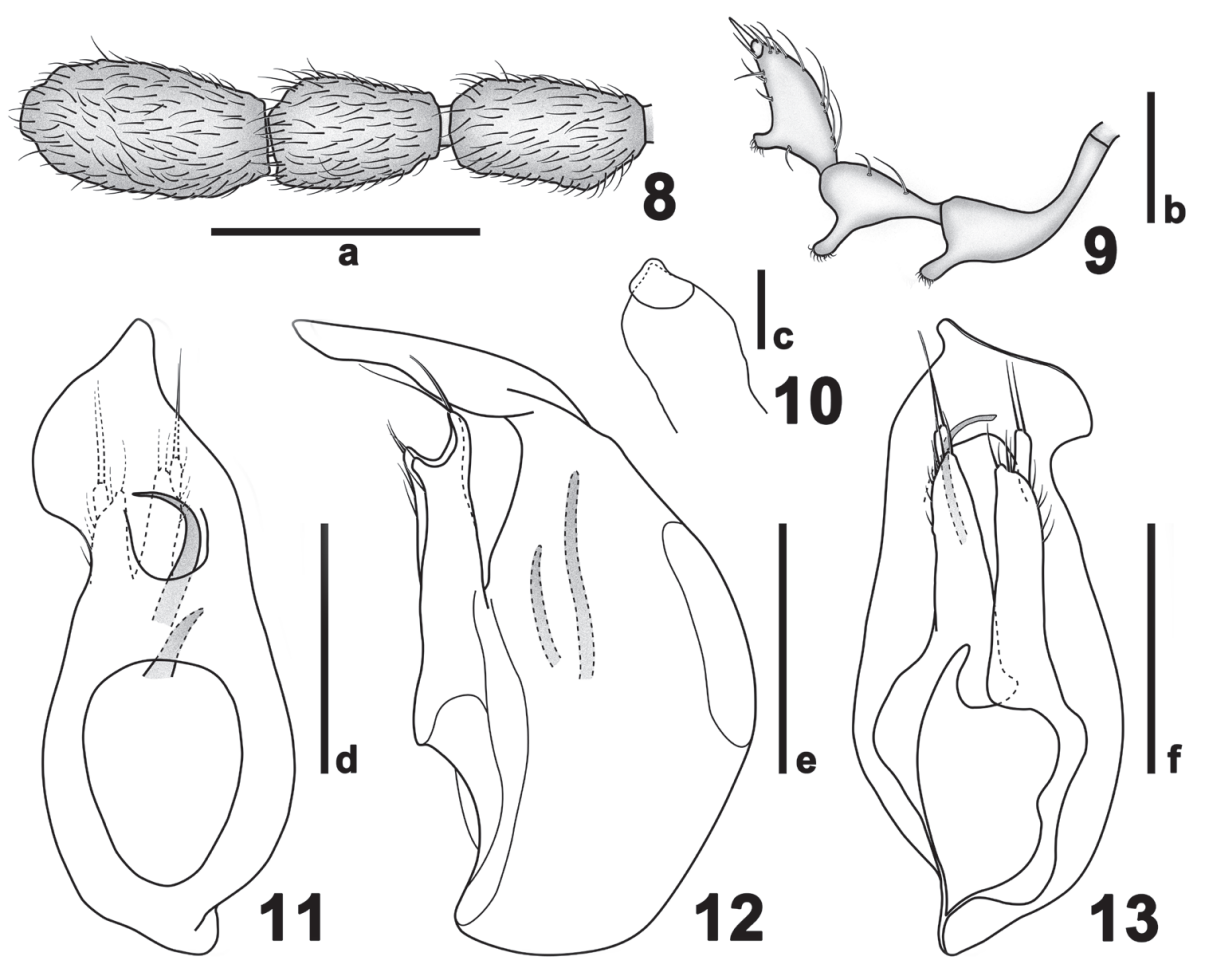
Details of *Dayao pengzhongi*. **8** male antennal club, enlarged **9** male left maxillary palpus **10** male sternite IX **11** aedeagus, in dorsal view **12** same, in lateral view **13** same, in ventral view. Scales: a = 0.3 mm, b–c = 0.1 mm, d–f = 0.2 mm.

Male (Fig. 1). Length 2.95 mm (holotype). Head lengthily and bluntly triangular, HL 0.74 mm, HW 0.59 mm, with decumbent setae. Vertexal foveae located posterior to point level with posterior margin of eyes. Eyes prominent, each with about 35 facets. Antennae elongate (Fig. 2), scape longer than II–IV combined, II–IV each short, of same width, V–VIII each longer than II–IV, slightly narrower, of same width, shortened distally, antennomeres of club (Fig. 8) of about same width, IX slightly expanded laterally at basal third, X shorter than IX and XI, XI longer than IX, with rounded apex. Pronotum (Fig. 4) as long as wide, PL 0.66 mm, PW 0.64 mm, with ‘Y’-shaped sulcus in anterior half, tufts of long golden setae at anterior margin of sulcus directed posteriorly. Elytra wider than long, EL 0.81 mm, EW 1.05 mm, lacking ridges. Venter (Fig. 6) with long metaventral process (Fig. 7) bent posteriorly near apex; metaventrite smooth, convex medially in apical half, with dense long setae at lateral portions and sparse minute setae at middle. Legs long, protibiae with small apical spines, pro- and mesofemora with distinct erect setae at ventral margins. Abdomen wider than long, AL 0.74 mm, AW 1.03 mm; discal carinae on tergite IV extending two-fifths of tergal length; tergite VIII transverse, narrowed apically in posterior half, with shallow median emargination, tergite IX (Fig. 10) semi-membranous, with apical portion strongly sclerotized; sternites VIII transverse. Aedeagus (Figs 11–13) length 0. 48 mm.

Female. In general similar to male. Antennomere IX (Fig. 3) and pronotum (Fig. 5) unmodified, metaventrite lacking process, protibiae simple. BL 2.81–2.99 mm, HL 0.71–0.73 mm, HW 0.57–0.59 mm, PL 0.63–0.66 mm, PW 0.62–0.64 mm, EL 0.74–0.78 mm, EW 1.07–1.10 mm, AL 0.73–0.82 mm, AW 1.12–1.15 mm. Eyes each with about 30 facets.

#### Etymology.

The specific name recognizes the efforts of Zhong Peng in collecting the male holotype.

## Supplementary Material

XML Treatment for Dayao

XML Treatment for Dayao pengzhongi
